# Perisomatic innervation on the semilunar granule cells and outer molecular layer granule cells of the dentate gyrus of the mouse

**DOI:** 10.3389/fnana.2026.1749335

**Published:** 2026-04-29

**Authors:** Laura Rovira-Esteban, Isaac Vieco-Martí, Javier Herraiz-Cabanes, Emilio Varea, Juan Nacher, Carlos Crespo, José Miguel Blasco-Ibáñez

**Affiliations:** 1 Neurobiology Unit, Institute for Biotechnology and Biomedicine (BIOTECMED), University of Valencia, Valencia, Spain; 2 CIBERSAM, Spanish National Network for Research in Mental Health, Madrid, Spain; 3 Institute of Research of the Clinic Hospital from Valencia (INCLIVA), Valencia, Spain

**Keywords:** dentate gyrus, electron microscopy, interneurons, mossy cells, semilunar cells, supramamillary nucleus (SUM)

## Abstract

Semilunar granule cells in the dentate gyrus represent a distinct type of granule cell, distinguished by their unique morphology and physiology. These cells are located within the inner molecular layer and the upper juxta-granule cell layer of the dentate gyrus. The presence of a smaller number of granule cells has also been observed in the outer molecular layer; however, the information regarding these cells is limited. In the present study, the perisomatic innervation that these two types of granule cells receive was characterized using confocal and electron microscopy. Our findings revealed that both semilunar granule cells and outer molecular layer granule cells receive substantial excitatory and inhibitory perisomatic contacts. The inhibitory contacts are derived from parvalbumin fast-spiking cells and cholecystokinin regular-spiking cells. The origin of excitatory contacts has been traced to hilar mossy cells and supramammillary afferents.

## Introduction

The dentate gyrus represents the gate of the input from the entorhinal cortex into the hippocampus. The axons from the entorhinal cortex (perforant pathway) contact the principal cells of the dentate gyrus, the granule cells, on their dendrites in the outer molecular layer. Granule cells in turn, send their axons, the mossy fibers, into the CA3 pyramidal cells. On their way to the CA3, the mossy fibers contact the principal cells of the hilus, the mossy cells, that in turn project back to the proximal segments of the granule cell dendrites in the inner molecular layer (Amaral et al., 2007).

The granule cells are small excitatory glutamatergic cells closely packed into the granule cell layer. The presence of granule cells with their somata located out of the granule cell layer has been known since early studies with the Golgi method (Ramón y Cajal, 1904), but they were considered misplaced granule cells, hence the name “ectopic cells.” The number of ectopic cells compared to normal granule cells is small. In the mouse, they represent at most 3% of the granule cells (Save et al., 2019), and most of them are located in the molecular layer of the dentate gyrus. Inside the molecular layer, most ectopic cells are located close to the upper side of the granule cell layer and in the inner molecular layer (semilunar granule cells), where they receive the contacts from the boutons of the hilar mossy cells onto their dendrites (Blasco-Ibáñez and Freund, 1997; Larimer and Strowbridge, 2010; Liu et al., 1996; Williams et al., 2007), although a few can be found in the outer molecular layer, where the dendrites of the granule cells are contacted by the perforant pathway. Ectopic granule cells can also be found in the hilus and even in the CA3 (Scharfman and Pierce, 2012); they are scarce in control animals but can increase in epileptic models, originating from postnatal neurogenesis (McCloskey et al., 2006). In this work, we will concern only about ectopic granule cells in the inner molecular layer (semilunar granule cells) and in the outer molecular layer, in control mice.

Molecular layer granule cells have received comparatively little attention (Sancho-Bielsa et al., 2012). The fact that they are scarce when compared to normal granule cells, but more importantly the lack of specific markers to distinguish them from normal granule cells has limited the depth of the study. In spite of that, in the last 20 years, they have been studied using whole cell patch clamp, allowing us to characterize the special relationship that they have with mossy cells with which they engage in reciprocal excitation known in hilar up-states (Larimer and Strowbridge, 2010; Williams et al., 2007). They have special electrophysiology and can be differentiated from granule cells by morphological criteria such as the number of dendrites arising from the soma (Gupta et al., 2020; Rovira-Esteban et al., 2020). Semilunar granule cells are also the origin of the perisomatic excitatory innervation of the parvalbumin cells of the granule cell layer by the granule cells (Blasco-Ibáñez et al., 2000; Rovira-Esteban et al., 2020). These studies have not been extended to outer molecular layer granule cells.

In the present study we aim to characterize, under confocal scanning microscopy and transmission electron microscopy, the perisomatic innervation received by the semilunar granule cells and outer molecular layer granule cells. In particular, the inhibitory innervation from parvalbumin fast spiking basket cells and cholecystokinin regular spiking basket cells, as well as the excitatory innervation proceeding from the hilar mossy cells and the supramammillary nucleus.

## Materials and methods

For this work we used Thy1 transgenic mice with C57BL/6J background (Strain name B6 Cg-Tg (Thy1-YFP) 16 Jrs/J. Ref: 003709, The Jackson Laboratory). Thy1 transgenic mice express the YFP protein under the Thy1 promoter, resulting in a selective staining of a discrete subpopulation of cortical principal neurons (Feng et al., 2000; Porrero et al., 2010). We also used sections from adult Wistar rats from previous experiments to test cholecystokinin staining. Nevertheless, all data for confocal scanning and electron microscopy used in this study came from the mice.

Adult animals included in this study were between 4 and 5 months old. No differentiation was made between males and females as there is no evidence of anatomical differences between them in the expression of any of the studied cell markers, or in the studied projections.

All animals were housed in groups of three to six under controlled temperature and humidity, and on a 12 h light/dark cycle, with access to food and water ad libitum. An effort was made to avoid unnecessary stress in the animals due to handling.

All animal experimentation was conducted in accordance with the Directive 2010/63/EU of the European Parliament and of the Council of 22 September 2010 on the protection of animals used for scientific purposes.

For the experiments of double immunofluorescence under confocal scanning microscopy four animals were perfused with 4% paraformaldehyde in 0.1 M pH 7.4 phosphate buffer (PB) with a 15% of a saturated solution of picric acid (30 min). For electron microscopy three different fixatives were used (two animals for each fixative): 0.5% glutaraldehyde, 4% paraformaldehyde in PB with a 15% of a saturated solution of picric acid (30 min); 1% glutaraldehyde, 4% paraformaldehyde in PB with a 15% of a saturated solution of picric acid (30 min); or 20 mL of 3.8% acrolein in 2% paraformaldehyde in PB (5 min) followed by 2% paraformaldehyde in PB (25 min). After perfusion, the brains were cut at 50 μm with a vibratome in six parallel series and stored with 0.05% sodium azide in PB at 4 °C until used.

For immunofluorescence, after adjusting the best concentrations for the antibodies, all the sections were processed simultaneously. Sections were processed free-floating in glass vials. They were blocked using 0.1% fish gelatin and 5% normal donkey serum (NDS) in PBS for 1 h. The primaries (see Table 1, for origin and concentration) were diluted in 1% NDS 0.1% Triton X-100 PBS. Sections were incubated in primaries for 5 days. Then, the sections were incubated in secondary antibody for 2 h (see Table 2). Sections were mounted on slides with Dako Mounting Medium (refractive index 1.47–1.5). The antibodies have been tested for specificity in their laboratories of origin, the labeling was consistent with the published labeling for these markers.

**Table 1:** Primary antibodies.

Antigen	Host	Dilution	Company	References
CB1r	rabbit	1:1000	Synaptic Systems	Cat. No. 258003
Calretinin	rabbit	1:2000	Swant	Cat. No. 7699/3H
Cholecystokinin 8	rabbit	1:1000	Sigma	Cat. No. C2581
pan-Fos (c-Fos K-25)	rabbit	1:1000	Santa Cruz Biotechnology	Cat. No. sc-253
Parvalbumin (25)	rabbit	1:2000	Swant	Cat. No. PV-28
Prox1	mouse	1:1000	Millipore	Cat. No. MAB5654
VGluT2	Guinea pig	1:1000	Millipore	Cat. No. AB2251

**Table 2:** Secondary antibodies.

Antibody	Host	Label	Dilution	Company
Anti-Rabbit IgG	Donkey	CF®555	1:200	Biotium
Anti-Rabbit IgG	Goat	Biotin	1:200	Vector
Anti-Mouse IgG	Goat	Biotin	1:200	Vector
Anti-Guinea Pig IgG	Donkey	CF®555	1:200	Biotium
Anti-Guinea Pig IgG	Donkey	Biotin	1:200	Jackson

For anterograde tracing from the supramammillary bodies into the dentate gyrus, we used iontophoretic injection of 10 kDa biotinylated dextran amine (BDA) into the supramammillary bodies using a stereotaxic frame at bregma −2.80 mm, lateral 0 mm, deep 4.3 mm (Paxinos and Franklin, 2012). The mice were sacrificed after 3 days, perfused with 0.5% glutaraldehyde, 4% paraformaldehyde in PB with a 15% of a saturated solution of picric acid (30 min), and cut at 50 μm with a vibratome. BDA was detected using ABC (Dako), and the coloring was developed using DAB intensified with nickel and H₂O₂ (black color). Selected sections were processed for electron microscopy (see below).

Cells were observed using a Leica TCS SPE confocal microscope. All the images were taken during two consecutive days using the same parameters of laser intensity, gain and offset. The cells were selected from dorsal dentate gyrus using conventional fluorescence with a 488 filter to detect Thy1-YFP labeled cells based on their location in the molecular layer and morphology (for juxta-granule cell layer semilunar granule cells). Then, a stack was acquired for the total depth of the cell with an HCX PL APO 63X NA 1.4 oil objective (1024×1024 pixels, pinhole 1 AU, zoom 4x, Z step size 0.3 μm, xy: 0.043 μm/pixel). Only cells whose whole soma was included between the limits of the stack were considered for the study. Boutons were counted manually using FIJI (Schindelin et al., 2012) with the help of Point Tool and Orthogonal Views. Only those puncta that overlapped the outside edge of the Thy1 somata with no apparent space between both profiles were considered positive. The data and graphics were processed using Microsoft Excel and JASP. Data are given as number of boutons per cell with standard deviation. Differences were tested using Student’s *t*-test, tested for Normality (Shapiro–Wilk test) and for Equality of Variances (Levene’s test). For representation we used box-and-whisker diagrams.

For electron microscopy, the sections were first treated with 1% sodium borohydride in PB for 10 min, then the sections were cryoprotected using 25% sucrose and 10% glycerol in PB 0.05 M; to enhance the penetration, the Freeze-Thawing technique was used (Blasco-Ibáñez and Freund, 1997). Then the sections were incubated in a blocking solution with 1% NDS in PB for 1 h and then in a mixture of primary antibodies (one for nucleus labeling and the other for the boutons) for 2 days (see Table 1). Then, we made a sequential labeling, first the boutons were labeled using biotinylated secondary antibody and then with ABC (Dako). The coloring was developed using DAB intensified with nickel and H_2_O_2_ (black color). Finally, the nuclei were visualized using the biotinylated secondary antibody and ABC, but this time the peroxidase was made evident using DAB and H_2_O_2_ (brown color).

The sections were treated with 1% osmium tetroxide and 1% uranyl acetate, dehydrated and then embedded in an epoxy resin (Durcupan) between slide and coverslip. Suitable candidates to be a semilunar or outer molecular layer granule cells were selected from dorsal hippocampus under light microscopy, the coverslip was removed and the cell excised with the help of a surgical blade and re-embedded for ultrathin cutting. 60 nm ultrathin sections were mounted on nickel slot grids covered with Formvar. Then, the sections were counterstained with lead citrate (Venable and Coggeshall, 1965) and uranyl acetate and observed under transmission electron microscopy with a JEOL jem 1,010 equipped with a digital camera AMT XR 80 with a CCD sensor of 8MPx. For the figures, the images from the electron microscope were false colored to make them as self-explanatory as possible.

## Results

**Figure 1:**
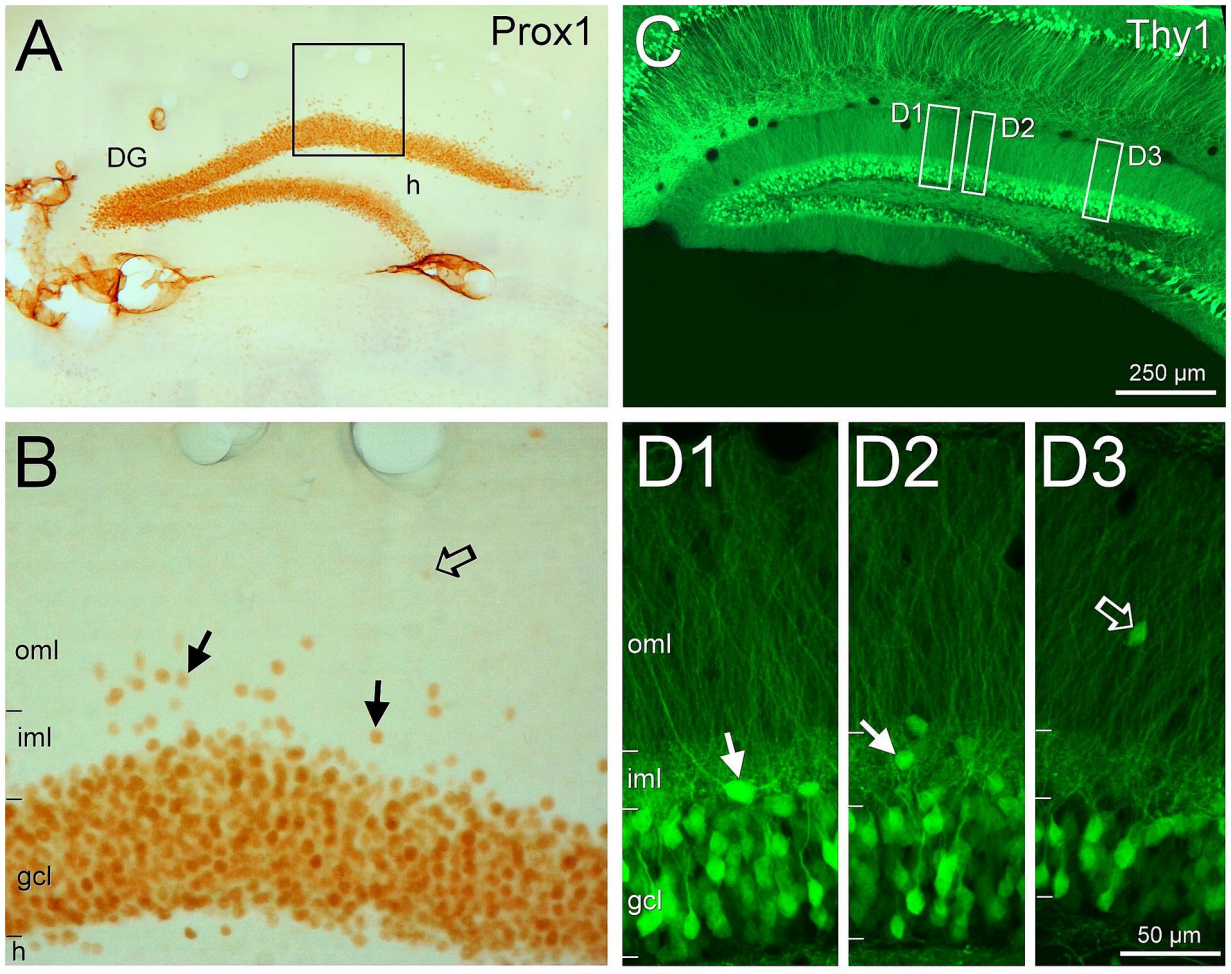
Ectopic granule cells in the molecular layer of the dentate gyrus. Prox1 labels all granule cells in the dentate gyrus **(A)**. The vast majority of granule cells have their somata in the granule cell layer but a few are present in the inner molecular layer (semilunar granule cells, arrows) and even in the outer molecular layer **(B**, open arrow**)**. Fluorescence under Thy1 is expressed by random principal cells, including granule cells **(C)**. The majority of the labeled granule cells are in the granule cell layer. However, semilunar granule cells adjacent to the granule cell layer but with horizontal soma shape and various dendrites arising from the soma **(D1**, arrow**)**, or higher in the inner molecular layer **(D2**, arrow**)** can also be found and, less abundantly, some ectopic granule cells in the outer molecular layer **(D3**, open arrow**)**. DG, dentate gyrus; h, hilus; GCL, granule cell layer; IML, inner molecular layer; OML, outer molecular layer. Scale bars: **A, C,** 250 μm; **B, D,** 50 μm.

Using immunoreactivity for Prox1 (prospero homeobox protein 1), a regulatory transcription factor expressed specifically by granule cells, numerous granule cells appeared in the molecular layer of the dentate gyrus. Most of them were located in the inner molecular layer, but a few were found in the outer molecular layer (Figures 1A,B). This is in accordance with other descriptions using Prox1 (Iwano et al., 2012). YFP expression under the Thy1 promoter labels principal cells in the cortex and hippocampus. In the dentate gyrus, it labels mature granule cells (Radic et al., 2015), but only a proportion of them (stochastically) are labeled (Figures 1C,D). The distributions of Prox1 and Thy1-positive granule cells in the molecular layer were similar, but Thy1 cells were scarcer than Prox1 cells since not all Prox1 cells were labeled for Thy1. A double labeling (Supplementary Figure 1) showed that Thy1 cells in the molecular layer co-expressed Prox1, confirming that they are granule cells.

To study the perisomatic innervation, different markers were used to label the target cells in light and electron microscopy. For light microscopy Thy1 was selected, since it labeled the cells Golgi-like. This allowed us to localize the boutons in apposition with the somata. Moreover, since the neuropil is also labeled, the inner molecular layer and outer molecular layer could be precisely delimitated (Figures 1C,D). For electron microscopy, Thy1 conversion to DAB was too dense and made identifying the boutons difficult. On the other hand, without labeling the granule cells, it was difficult to locate the baskets of boutons innervating perisomatically the cells. Because of this, nuclear labeling was used to locate candidate granule cells under light microscopy, but then, we always used the ultrastructure of the cells to confirm their identity (Lübbers and Frotscher, 1987; Shapiro and Ribak, 2005). We used two approaches, with either Prox1 or Fos, to evidence granule cells. Both rendered nuclear labeling, allowing to locate the cells without interfering with the bouton visualization. Prox1 is specific to granule cells, but the labeling and penetration were sometimes weak in the material for electron microscopy. In the dentate gyrus, Fos is mainly expressed by principal cells under normal conditions (Domínguez et al., 2003). The labeling with Fos was stronger, but since not all cells were Fos positive the number of possible targets diminished. We used both approaches, selecting for each combination the best possible candidate from the different fixatives. For calretinin and vesicular glutamate transporter 2 (Vglut2) boutons we decided on Prox1 as nuclear labeling, whereas for parvalbumin and cannabinoid receptor 1 (CB1r) we preferred Fos.

### Perisomatic innervation on semilunar granule cells

**Figure 2:**
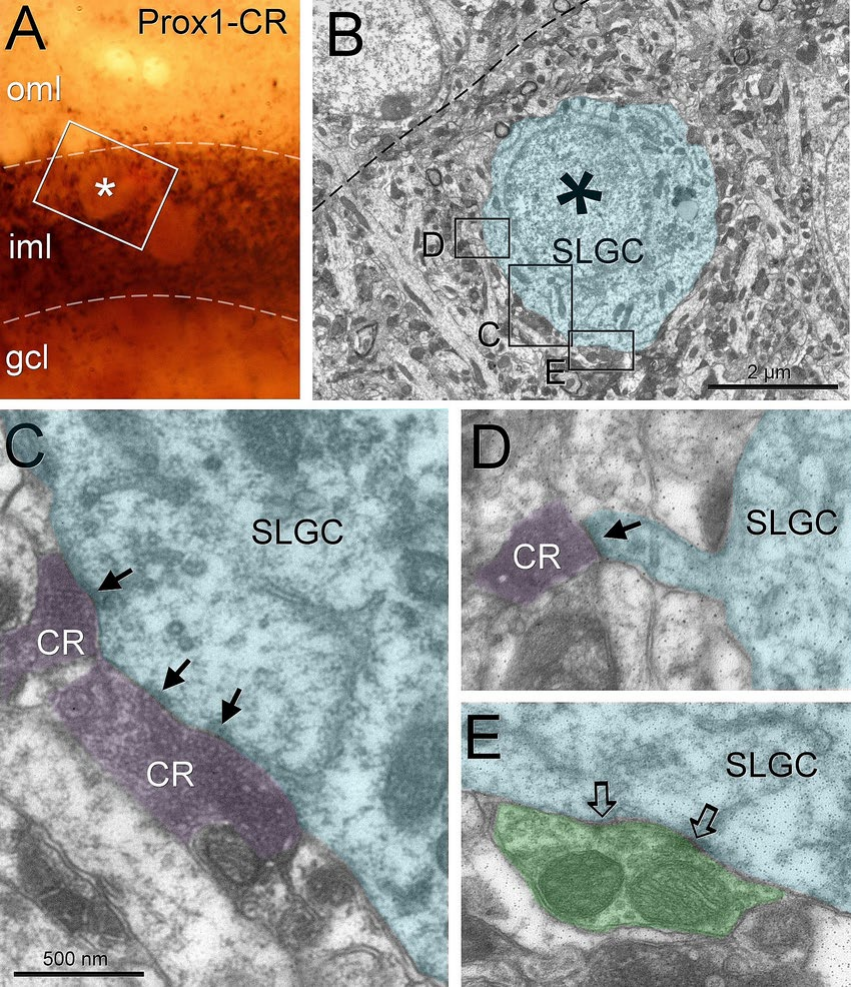
Semilunar granule cells are perisomatically innervated by hilar mossy cells. Semilunar granule cells (labeled with Prox1) are surrounded by hilar mossy cell boutons (calretinin-positive) in the inner molecular layer **(A)**. The cell labeled with an asterisk was sectioned for electron microscopy **(B)**. Hilar mossy cell boutons made asymmetric contacts **(C,D**, arrows**)** on the soma and a somatic spine, respectively. On the same ultrathin section, an unlabeled bouton likely corresponding to a parvalbumin basket cell, made a symmetric contact (open arrows) on the soma **(E)**. Blue toned, semilunar granule cell, magenta toned, hilar mossy cell boutons, green toned, unlabeled inhibitory bouton. CR, calretinin; GCL, granule cell layer; IML, inner molecular layer; OML, outer molecular layer; SLGC, semilunar granule cell. Scale bars: **B**, 2 μm; **C–E**, 500 nm.

To identify the projection from the hilar mossy cells we used calretinin, that in mouse labels this projection (Blasco-Ibáñez and Freund, 1997; Liu et al., 1996). Semilunar granule cells were surrounded by calretinin boutons in the inner molecular layer of mice. Under electron microscopy, we could confirm that these cells received asymmetrical contacts from calretinin boutons (Figure 2). Some of these boutons made contact on somatic spines (Figure 2D). In rats, calretinin labels boutons coming from the supramammillary nucleus (Maglóczky et al., 1994), therefore we needed to exclude this possibility to use calretinin as a label for mossy cell boutons in mice. A double staining for Vglut2 and calretinin (Supplementary Figure 2) showed that whereas Vglut2 and calretinin co-localized in the inner molecular boutons in rats, they did not co-localize in mice.

**Figure 3:**
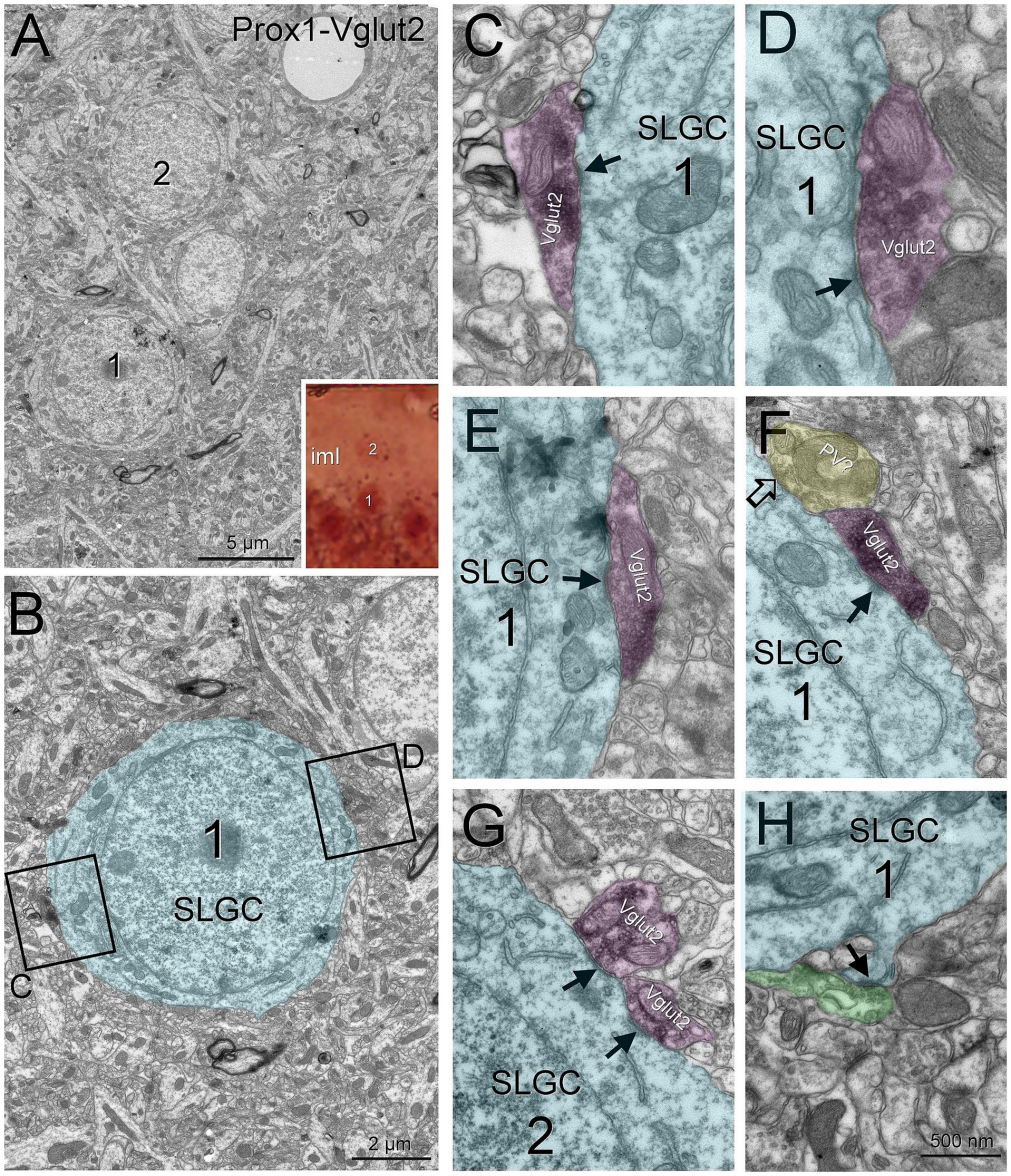
Semilunar granule cells are perisomatically innervated by supramammillary afferents. Semilunar granule cells (labeled with Prox1) are surrounded by puncta labeled for Vglut2 from the supramammillary nucleus **(A)**. The cells were studied under electron microscopy **(A,B)**. Supramammillary boutons making asymmetric contacts (arrows) were found on both cells **(C–G)**. Additionally, we found unlabeled boutons making symmetric contacts **(F**, open arrow, likely a parvalbumin bouton**)** as well as an asymmetric contact on a spine **(H**, solid arrow, likely a hilar mossy cell bouton**)**. Blue toned, semilunar granule cell; magenta toned, supramammillary boutons; green toned, unlabeled excitatory bouton; yellow toned, unlabeled inhibitory bouton. Iml, inner molecular layer; SLGC, semilunar granule cell. Scale bars: **B**, 2 μm; **C–H**, 500 nm.

To identify the projection from the supramammillary nucleus, we used Vglut2 (Boulland et al., 2009; Soussi et al., 2010). Semilunar granule cells were surrounded by Vglut2 boutons (Figure 3). Electron microscopy showed these boutons making asymmetric contacts on the semilunar granule cells. Somatic spines on semilunar granule cells were not contacted by Vglut2 boutons but by unlabeled boutons making asymmetric contacts (likely from the mossy cells). To confirm that the Vglut2 boutons we observed come from the supramammillary nucleus, we performed a 10 kDa BDA anterograde tracing experiment. We found BDA fibers coming from the supramammillary nucleus contacting semilunar granule cells in the inner molecular layer and outer molecular layer granule cells and confirmed the asymmetrical contacts under electron microscopy (Supplementary Figure 3). These results confirmed the adequacy of using Vglut2 as supramammillary nucleus bouton label in the dentate gyrus.

**Figure 4:**
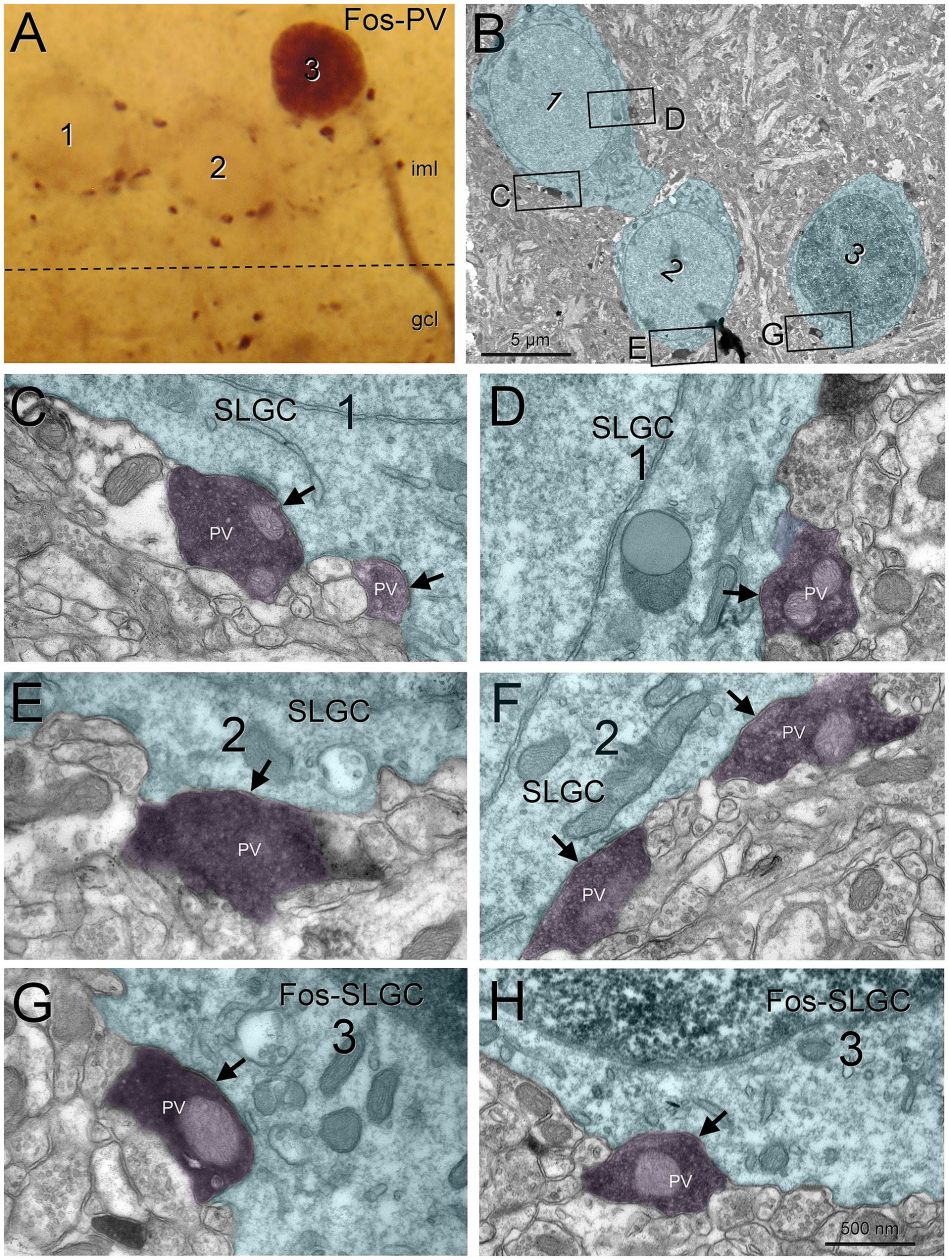
Semilunar granule cells are perisomatically innervated by fast spiking basket cells. Three semilunar granule cells in the iml, one of them labeled for Fos **(A)** were studied under electron microscopy **(B)**. All of them were surrounded by parvalbumin boutons making symmetric synapses **(C–H**, arrows**)**. Blue toned, semilunar granule cell; magenta toned, parvalbumin boutons. Gcl, granule cell layer; iml, inner molecular layer; PV, parvalbumin; SLGC, semilunar granule cell. Scale bars: **B**, 1 μm; **C–H**, 500 nm.

Semilunar granule cells were also contacted by fast-spiking basket cells. Using parvalbumin as marker for fast-spiking basket cells (Kawaguchi et al., 1987), we found that semilunar granule cells received symmetrical contacts from parvalbumin boutons. These boutons were often large and had abundant mitochondria (Figure 4).

**Figure 5:**
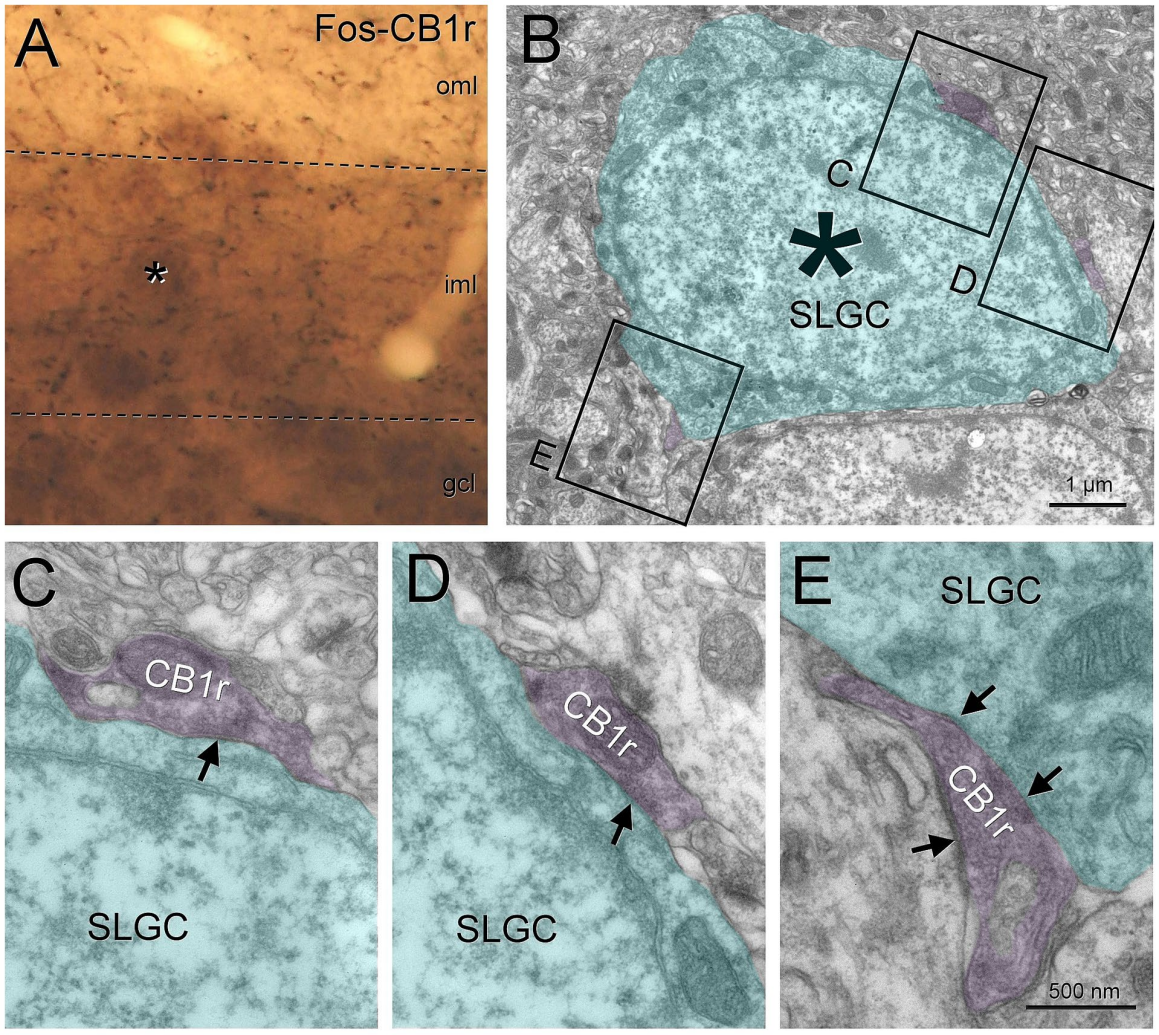
Semilunar granule cells are perisomatically innervated by regular spiking basket cells. A semilunar granule cell in the inner molecular layer labeled with Fos (asterisk), was selected to check if the regular spiking basket boutons (labeled with CB1r) made perisomatic contacts **(A)**. When visualized under electron microscopy **(B)**, we found that the CB1r boutons make symmetrical contacts (arrows) on the soma **(C–E)**. Blue toned, semilunar granule cell; magenta toned, CB1r boutons. Gcl, granule cell layer; iml, inner molecular layer; oml, outer molecular layer; SLGC, semilunar granule cell. Scale bars: **B**, 5 μm; **C–E**, 500 nm.

Finally, we also found evidence of semilunar granule cells being perisomatically contacted by regular spiking basket cells. We tested cholecystokinin (Pawelzik et al., 2002) and CB1r as markers for these boutons. In rat, the labeling for cholecystokinin was stronger and we found boutons making symmetric synaptic contacts on the molecular layer granule cells (Supplementary Figure 4), but in mouse the cholecystokinin labeling was weaker and we decided for CB1r. We found putative baskets surrounding the semilunar granule cells and correlation with electron microscopy showed CB1r boutons making symmetric contacts on semilunar granule cells in mouse (Figure 5), confirming the results for cholecystokinin that we found in rat.

### Perisomatic innervation on outer molecular layer granule cells

**Figure 6:**
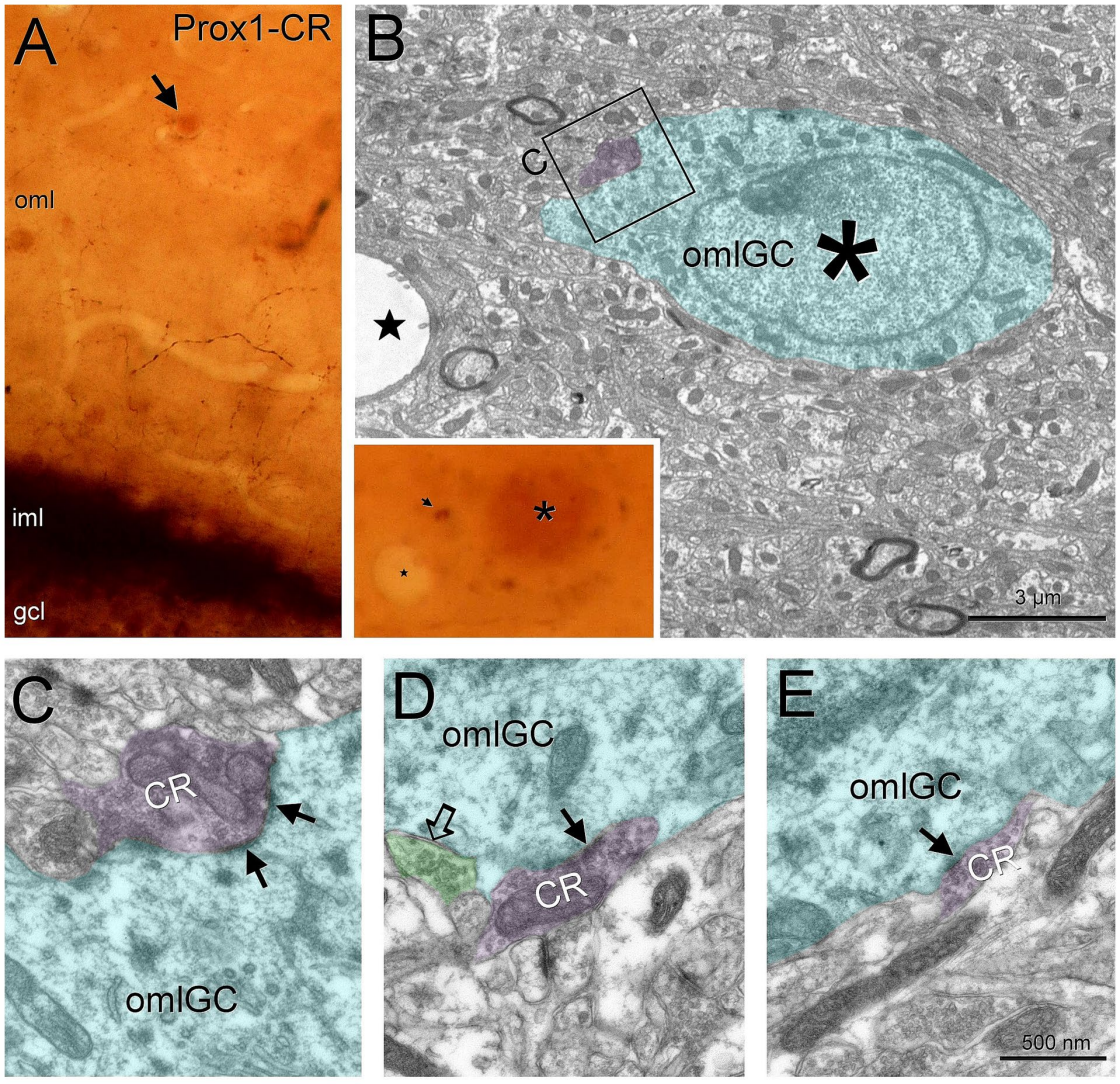
Outer molecular layer granule cells are perisomatically innervated by hilar mossy cells. Outer molecular layer granule cells (arrow, labeled with Prox1) are surrounded by calretinin puncta **(A)**. Under electron microscopy we observed that the correlated cell **(B**, asterisk**)** is contacted by calretinin boutons from the mossy cells making asymmetrical synapses **(C–E**, solid arrows**)**. We also observed an unlabeled bouton making a symmetrical synapse on the soma **(D**, open arrow**)**. Blue toned, outer molecular layer granule cell, magenta toned, hilar mossy cell boutons, green toned, unlabeled inhibitory bouton. Gcl, granule cell layer; iml, inner molecular layer; oml, outer molecular layer; omlGC, outer molecular layer granule cell. Scale bars: **B**, 3 μm; **C–E**, 500 nm.

When calretinin was used to label the axons from the mossy cells, we found isolated fibers in the outer molecular layer. Using Prox1 as a nuclear marker, we found baskets of calretinin boutons around these nuclei (Figure 6A). Correlation with electron microscopy showed that those boutons made asymmetric contacts with granule cells in the outer molecular layer (Figures 6B–E).

**Figure 7:**
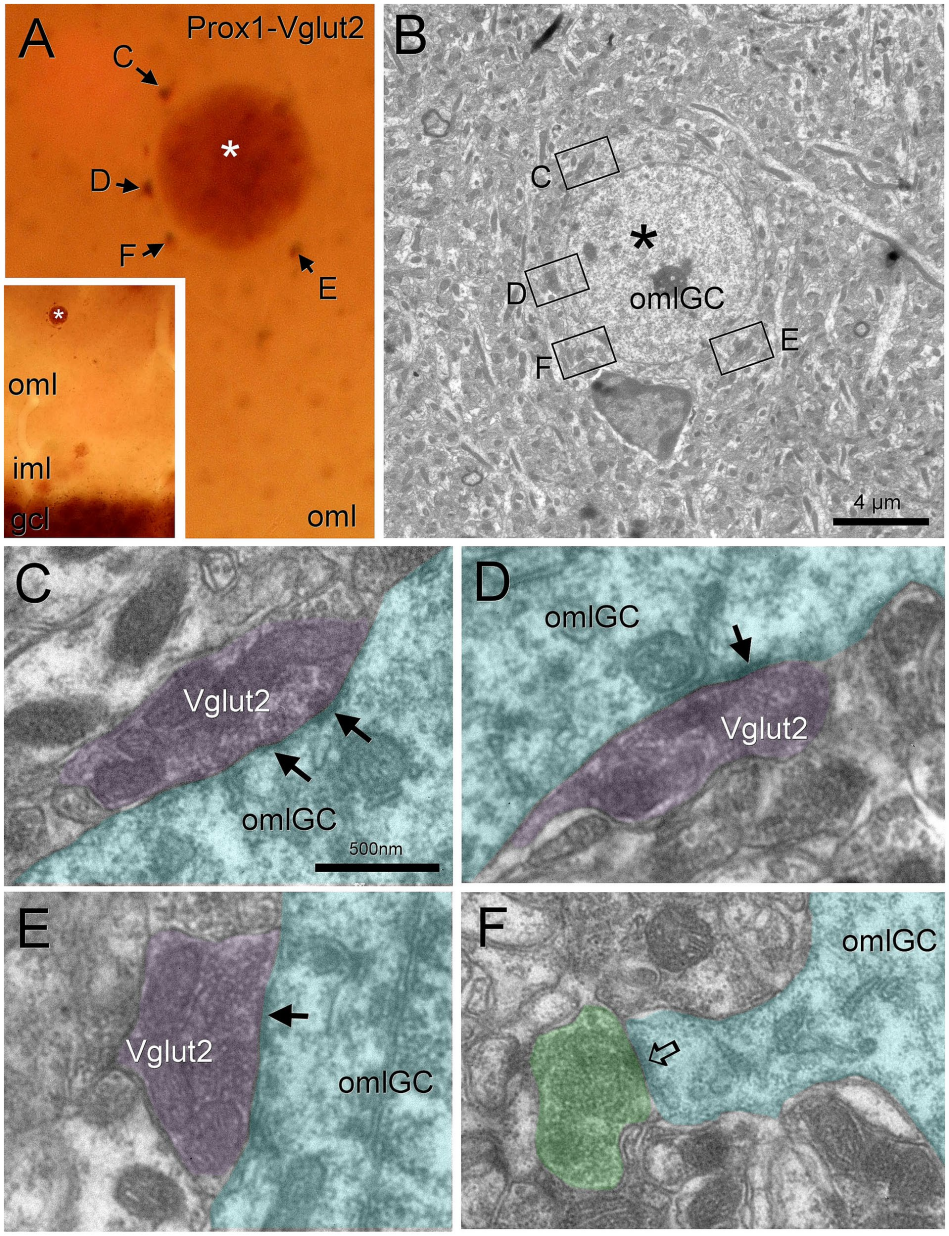
Outer molecular layer granule cells are perisomatically innervated by supramammillary afferents. Outer molecular layer granule cells (asterisk, labeled with Prox1) are surrounded by Vglut2 puncta **(A**, arrows**)**. Under electron microscopy **(B)**, we found that these boutons made asymmetrical contacts on the soma **(C–E**, arrows**)**. We also observed an unlabeled bouton (likely from a hilar mossy cell) making an asymmetric synapse on a well-developed somatic spine **(F**, open arrow**)**. Blue toned, outer molecular layer granule cell; magenta toned, supramammillary boutons; green toned, unlabeled excitatory bouton; yellow toned, unlabeled inhibitory bouton. Iml, inner molecular layer; oml, outer molecular layer; omlGG, outer molecular layer granule cell. Scale bars: **B**, 4 μm; **C–F**, 500 nm.

In a similar way, we found Vglut2 boutons from the supramammillary nucleus surrounding Prox1 nuclei in the outer molecular layer (Figure 7A). These boutons made asymmetric contacts on outer molecular layer granule cells (Figures 7B–E). The granule cell of this layer also presented well developed somatic spines, that received asymmetrical contacts from Vglut2 immunonegative boutons making asymmetrical contacts (Figure 7F).

**Figure 8:**
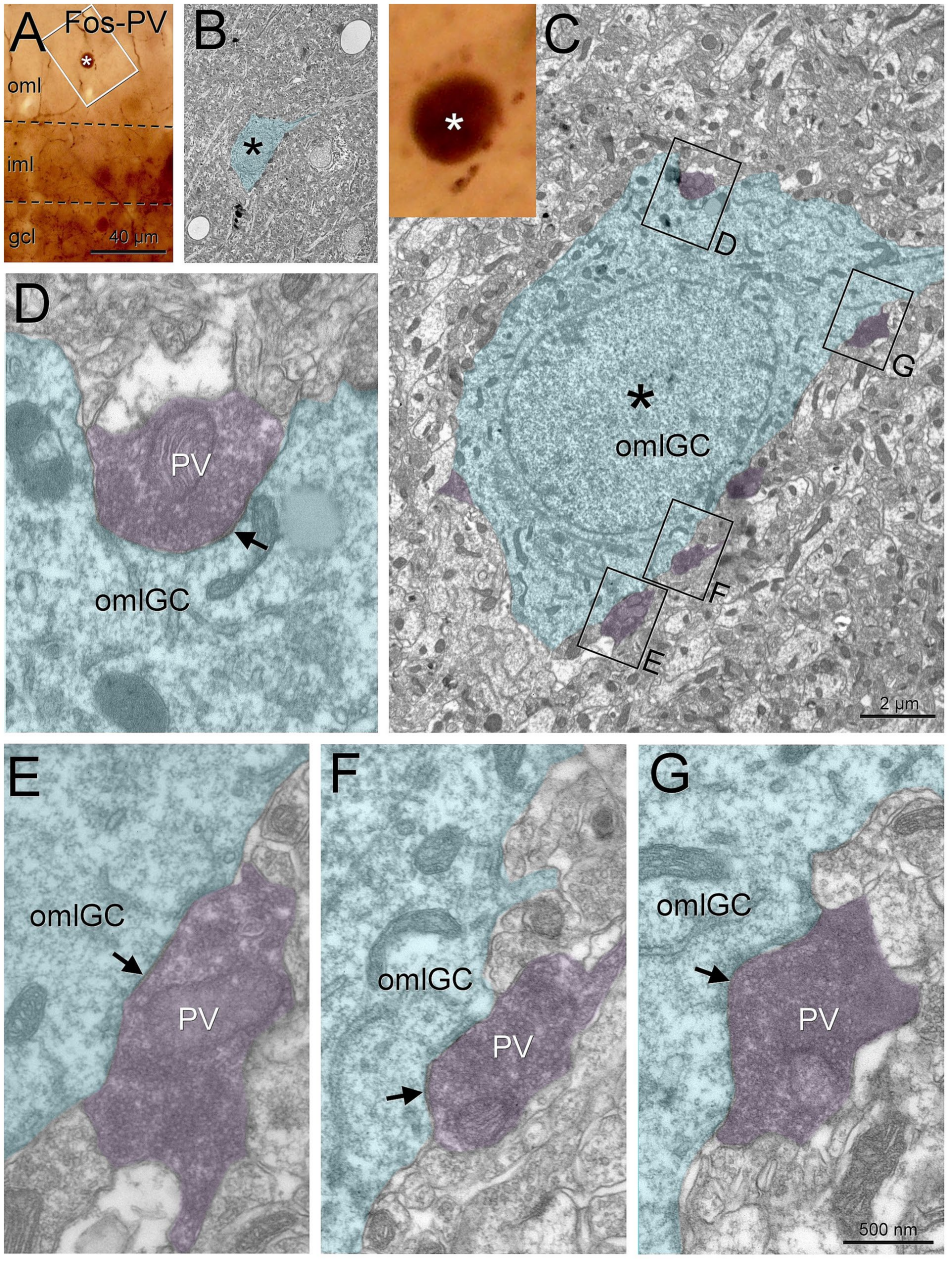
Outer molecular layer granule cells are perisomatically innervated by fast spiking basket cells. Outer molecular layer granule cells (asterisk, labeled with Fos) are surrounded by parvalbumin puncta **(A,B)**. Under electron microscopy **(C)**, we found that these boutons made symmetrical contacts on the soma **(D–G**, arrows**)**. Blue toned, outer molecular layer granule cell; magenta toned, parvalbumin boutons. GCL, granule cell layer; IML, inner molecular layer; OML, outer molecular layer; OMLGG, outer molecular layer granule cell. Scale bars: **A**, 40 μm; **B**, 2 μm; **D–G**, 500 nm.

We also found that the parvalbumin boutons from fast spiking basket cells contacted the granule cells in the outer molecular layer (Figures 8A,B). We used Fos to make evident the granule cells. The parvalbumin boutons were large and made symmetrical contacts on the granule cells (Figures 8C–G).

**Figure 9:**
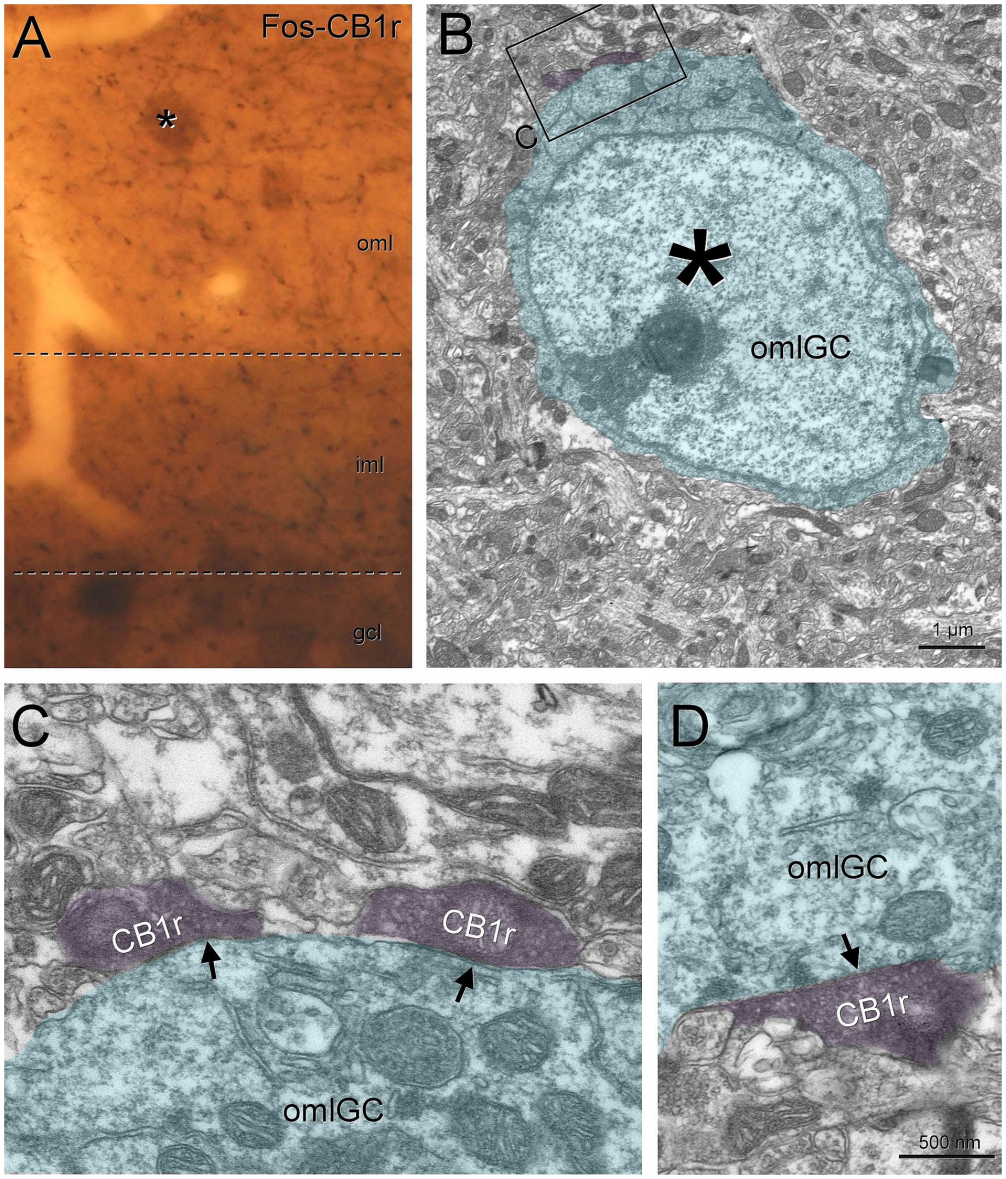
Outer molecular layer granule cells are perisomatically innervated by regular spiking basket cells. Outer molecular layer granule cells (asterisk, labeled with Fos) are surrounded by CB1r puncta **(A,B)**. Under electron microscopy **(C)** we found that these boutons made symmetrical contacts on the soma **(D–G**, arrows**)**. Blue toned, outer molecular layer granule cell; magenta toned, CB1r boutons. GCL, granule cell layer; IML, inner molecular layer; OML, outer molecular layer; OMLGG, outer molecular layer granule cell. Scale bars: **B**, 1 μm; **C, D**,500 nm.

Finally, we also found that the CB1r boutons from regular spiking basket cells contact the soma of the outer molecular layer granule cells (Figure 9A). Using Fos to correlate the light and electron microscopy, we found that these CB1r boutons made symmetrical synaptic contacts on these granule cells (Figures 9B–D).

### Frequency of contacts on semilunar granule cells

**Figure 10:**
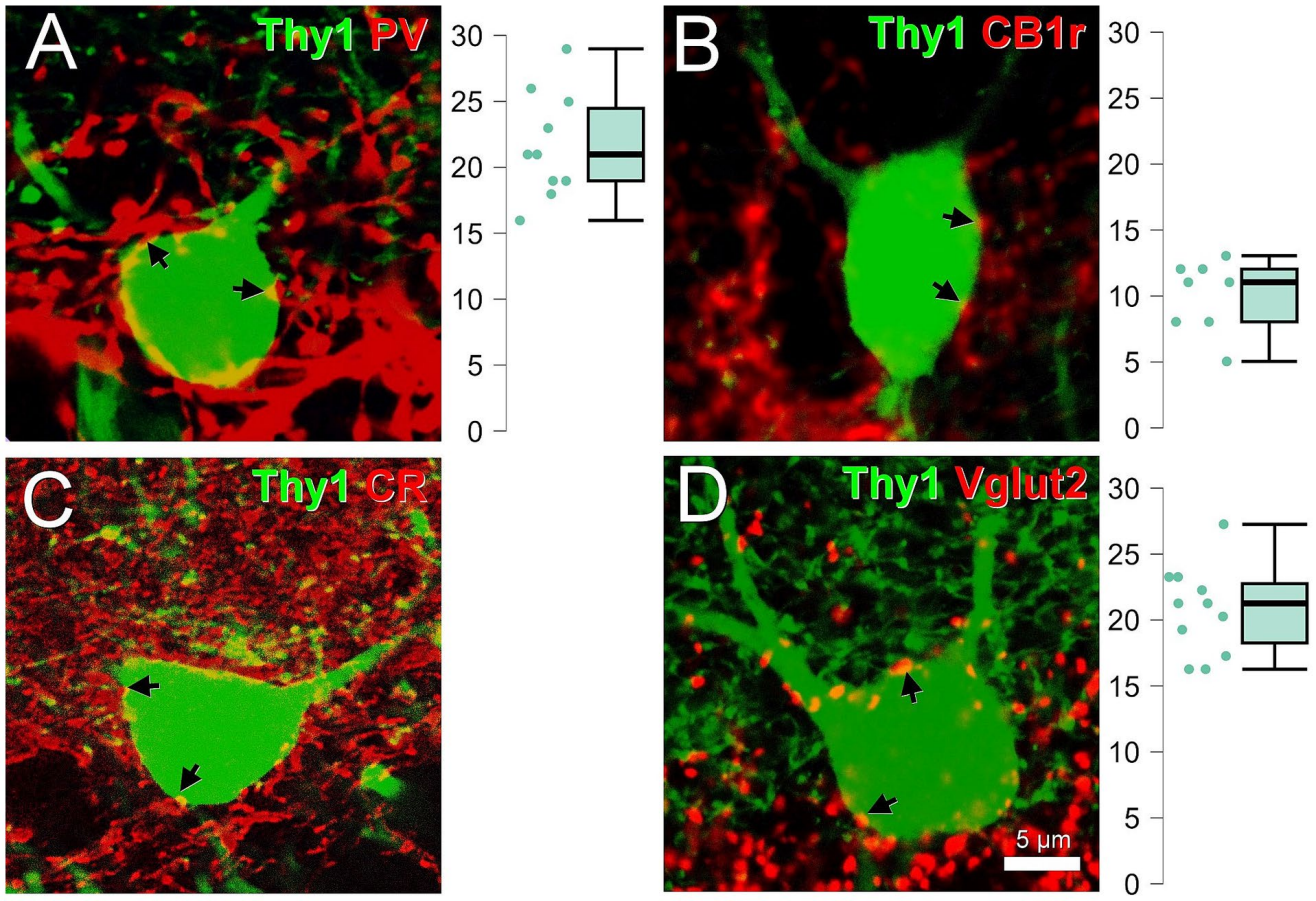
Perisomatic innervation on semilunar granule cells. Thy1 semilunar granule cells in the inner molecular layer are surrounded by parvalbumin **(A)**, CB1r **(B)**, calretinin **(C)**, and Vglut2 **(D)**. The arrows labsssel some of the boutons in apposition with the cells. Plots showing the number of boutons per cell are shown next to them. All images are single confocal planes. Image **C** was deconvoluted using DeconvolutionLab 2 (Sage et al., 2017) due to the large number of calretinin puncta in the inner molecular layer. For this marker, the number of puncta could not be estimated. All images are single confocal planes. Scale bar, 5 μm.

Under the confocal scanning microscope, we evaluated the frequency of boutons for each presynaptic marker on Thy1 fluorescent semilunar granule cells (Figure 10). The fluorescence labeled the cells Golgi-like and allowed us to visualize their size and their morphological distinctive characteristics. This was relevant to distinguish juxta-granular semilunar granule cells from normal granule cells in the juxta-granular upper border of the inner molecular layer. Semilunar granule cells had more than one dendrite arising from the soma and their dendrites spread in a rather obtuse angle. Their somata were large for granule cells 11.42 μm wide (SD 1.38, *n* = 31), 11.83 μm high (SD 1.82) and their aspect ratio was 1.06 (SD 0.243). We found that they were frequently innervated: for parvalbumin, 21.7 boutons per cell (SD 4.03, *n* = 10); for CB1r, 10.0 boutons per cell (SD 2.73, *n* = 10); for Vglut2, 20.45 boutons per cell (SD 3.36, *n* = 11). For calretinin, we could not evaluate the number of boutons. The calretinin boutons are small and densely packed (as confirmed by the electron microscopy) and most of the boutons in the inner molecular layer are calretinin immunoreactive (Blasco-Ibáñez and Freund, 1997). Even after deconvolution (Figure 10C) we could not estimate the number of boutons on these cells.

### Frequency of contacts on the outer molecular layer granule cells

**Figure 11:**
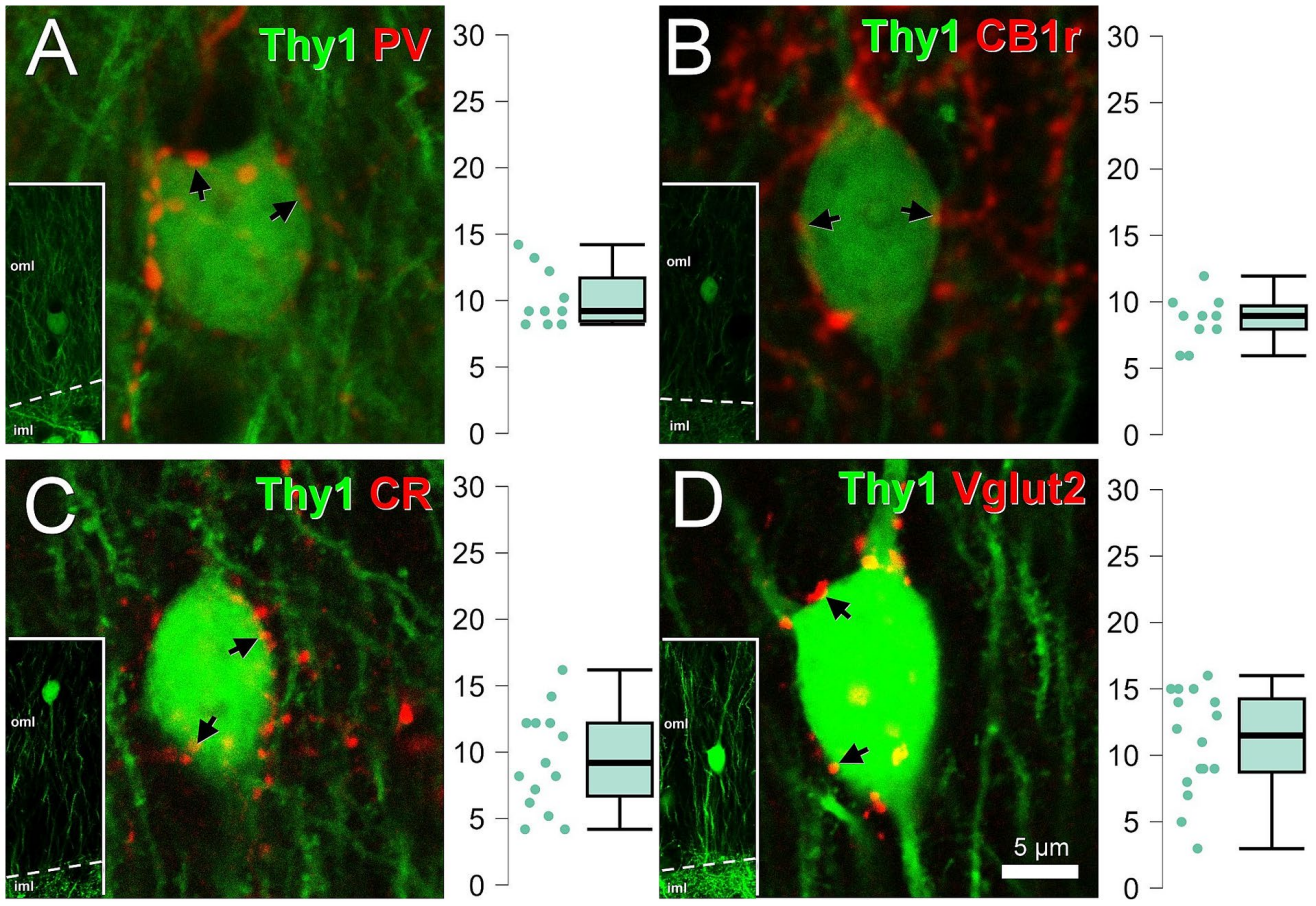
Perisomatic innervation on outer molecular layer granule cells. Thy1 cells in the outer molecular layer surrounded by parvalbumin **(A)**, CB1r **(B)**, calretinin **(C)**, and Vglut2 **(D)**. The arrows label some of the boutons in apposition with the cells. Plots showing the number of boutons per cell are shown next to them. Insets show the location of the soma in the outer molecular layer. All images are single confocal planes. Iml, inner molecular layer; oml, outer molecular layer. Scale bar, 5 μm.

Outer molecular layer granule cells (Figure 11), had a soma size of 9.52 μm wide (SD 0.99, *n* = 41), 14.54 μm high (SD 1.83) their aspect ratio was 1.54 (SD 0.19), they often had more than one dendrite arising from the apical part of the soma but unipolar cells were also present (Figure 11). The spread of their dendritic arbor was not as obtuse as in semilunar granule cells. They also received the same types of boutons on the surface of the soma, although they are less abundant than in the semilunar granule cells: for parvalbumin, 10.00 boutons per cell (SD 2.21, *n* = 10); for CB1r, 8.70 boutons per cell (SD 1.83, *n* = 10); for calretinin, 9.33 boutons per cell (SD 3.71, *n* = 15) and for Vglut2, 10.94 boutons per cell (SD 3.94, *n* = 17) (Figures 11A–D).

### Differences in innervation between semilunar granule cells and outer molecular layer granule cells

Semilunar granule cells received more parvalbumin and Vglut2 boutons than outer molecular layer granule cells (*p* < 0.001); for CB1r boutons the difference was not significant (*p* = 0.122); for calretinin it could not be tested. The outer molecular layer granule cells were more elongated in the vertical axis than the semilunar granule cells (*p* < 0.001).

## Discussion

The main finding of this work is that both semilunar granule cells and outer molecular layer granule cells are perisomatically innervated by mossy cells, supramammillary nucleus, fast spiking basket cells and regular spiking basket cells. These cells sometimes present somatic spines that are innervated by mossy cell axons.

### Perisomatic innervation of semilunar granule cells

Semilunar granule cells have an important role in the function of the dentate gyrus. They are persistently depolarized after a transient input and regulate the gating of information through the hippocampus (Walker et al., 2010). Nrg1 nuclear back signaling is responsible for their differentiation into semilunar granule cells (Rajebhosale et al., 2025). They integrate the information in a different way from other types of granule cells (Mao et al., 2025) with preferential semilunar granule cell recruitment to contextual memory engrams (Dovek et al., 2024). Semilunar granule cells express Prox1, but are inherently different in inhibition from granule cells (Gupta et al., 2012) and maintain higher spontaneous inhibitory postsynaptic current (sIPSC) frequency than GC (Gupta et al., 2020, 2022).

Our results indicate that semilunar granule cells have an abundant perisomatic innervation from excitatory cells in addition to inhibitory basket cells. Since perisomatic innervation is in a position to heavily influence the excitability of a neuron as an on/off switch by powerfully and rapidly suppressing or facilitating firing, it can sharpen the temporal integration of synaptic inputs.

Inhibitory basket cells can be further subdivided into two types, fast spiking basket cells and regular spiking basket cells (Freund and Katona, 2007). For fast spiking basket cells the canonical marker is parvalbumin (Kawaguchi et al., 1987) whereas for regular spiking basket cells, although they were first studied using cholecystokinin (Pawelzik et al., 2002), they can also be studied using CB1r (Tsou et al., 1999). In mice, we decided to use CB1r since the labeling and the contrast were higher. The number of parvalbumin boutons on semilunar granule cells was about double the number of CB1r, although in the dentate gyrus the number of parvalbumin and cholecystokinin is about the same (Whissell et al., 2015). This imbalance between the two types of perisomatic inhibition can suggest to a larger involvement of semilunar granule cells in synchronizing effects rather than in synaptic modulation (Freund, 2003). The relevance of parvalbumin innervation on semilunar granule cells has been confirmed using miniature inhibitory potentials (Afrasiabi et al., 2022). Our anatomical data agree with those observations and confirm the relevance of this innervation. It should be considered that semilunar granule cells specifically target parvalbumin basket cells in the granule cell layer (Rovira-Esteban et al., 2020). The feedback that semilunar granule cells receive from the parvalbumin basket cells confirms the strength of this inhibitory loop.

Semilunar granule cells were also perisomatically innervated by mossy cell axons. These axons are glutamatergic excitatory boutons. Although they could not be properly quantified, they were abundant. The existence of these boutons on the soma is striking. Both normal granule cells and semilunar granule cells receive mossy cell boutons in the dendrites in the inner molecular layer (mostly on spines; data not shown). It seems unnecessary for them to receive additional excitation on the soma membrane. Semilunar granule cells are known to engage with hilar mossy cells in an excitatory loop known as hilar up-states (Williams et al., 2007). Larimer and Strowbridge (2010) described that this differential behavior from normal mossy cells was not due to the recurrent connectivity but rather to different intrinsic properties of the semilunar granule cells, among them a dissimilar expression of voltage gated calcium channels. Semilunar granule cells and mossy cells originate from the earliest stages of developmental neurogenesis and those early born neurons form age-matched circuits with each other (Save et al., 2019). Semilunar granule cells are qualitatively different from normal granule cells due to the presence of synapses from mossy cells on the soma and, sometimes, on somatic spines. Mossy cells are necessary for the function of granule cells (Jinde et al., 2012, 2013), but in semilunar granule cells, they appear to have even higher relevance.

Supramammillary fibers also extensively innervate the semilunar granule cells perisomatically. The density of boutons on semilunar granule cells was elevated, similar to the quantity of boutons from parvalbumin cells. Supramammillary innervation plays a significant role in the dentate gyrus. It stimulates a group of granule cells and enhances theta and gamma activity during paradoxical sleep. It is essential for spatial learning and may regulate hippocampal plasticity (Billwiller et al., 2020; Kirk, 1998; Pan and McNaughton, 1997, 2004). It plays an essential role in various behavioral and cognitive functions, such as reward-seeking, exploration, and social memory, and is vital in hippocampal plasticity and neurogenesis (Kesner et al., 2023). The supramammillary nucleus not only controls fundamental locomotor behavior but also selectively influences hippocampal neural activity in a way that might aid spatial navigation (Farrell et al., 2021). Theta rhythm is synchronized in the supramammillary nucleus and the dentate gyrus (Kirk and McNaughton, 1991). The circuit between the supramammillary region and the dentate gyrus plays a crucial role in creating and recalling spatial memories, with the glutamatergic transmission from the supramammillary region being essential for the strong correlation of activity between these two areas during memory retrieval (Li et al., 2020).

The innervation from the supramammillary nucleus to the dentate gyrus is both inhibitory and excitatory, although the combined result is excitatory (Hashimotodani et al., 2018). Because these boutons create a conductance for Cl− and Na+, their impact would vary based on the membrane potential of the semilunar granule cells. The innervation of the supramammillary fibers has a facilitating effect in the perforant path-evoked population spike in the dentate gyrus (Carre and Harley, 1991). Electrical stimulation of the supramammillary nucleus induces LTP at the perforant path granule cell synapses (Nakanishi et al., 2001). After LTP, the supramammillary inputs became sufficient to discharge granule cells (Tabuchi et al., 2022). We observed using anterograde tracing that usually a single fiber from the supramammillary nucleus produces three to four boutons on the target semilunar granule cell, therefore increasing the effect on the depolarization of the cells. This facilitating effect can be mediated by the semilunar granule cells that would induce the hilar up-states when entorhinal firing recruits the other granule cells. Due to the different electrophysiology of normal granule cells and semilunar granule cells, the supramammillary perisomatic innervation can be the switch that couples the theta phase of the supramammillary nucleus and the dentate gyrus (Kirk and McNaughton, 1991), controlling the passage of the information from the entorhinal cortex through the dentate gyrus. The mossy cell fibers innervate the semilunar granule cells, forming an intrinsic connection. Although they can induce hilar up states, they require regulation by an extra-hippocampal connection. The supramammillary nucleus’s innervation of semilunar granule cells is perfectly situated to offer this regulation.

**Figure 12:**
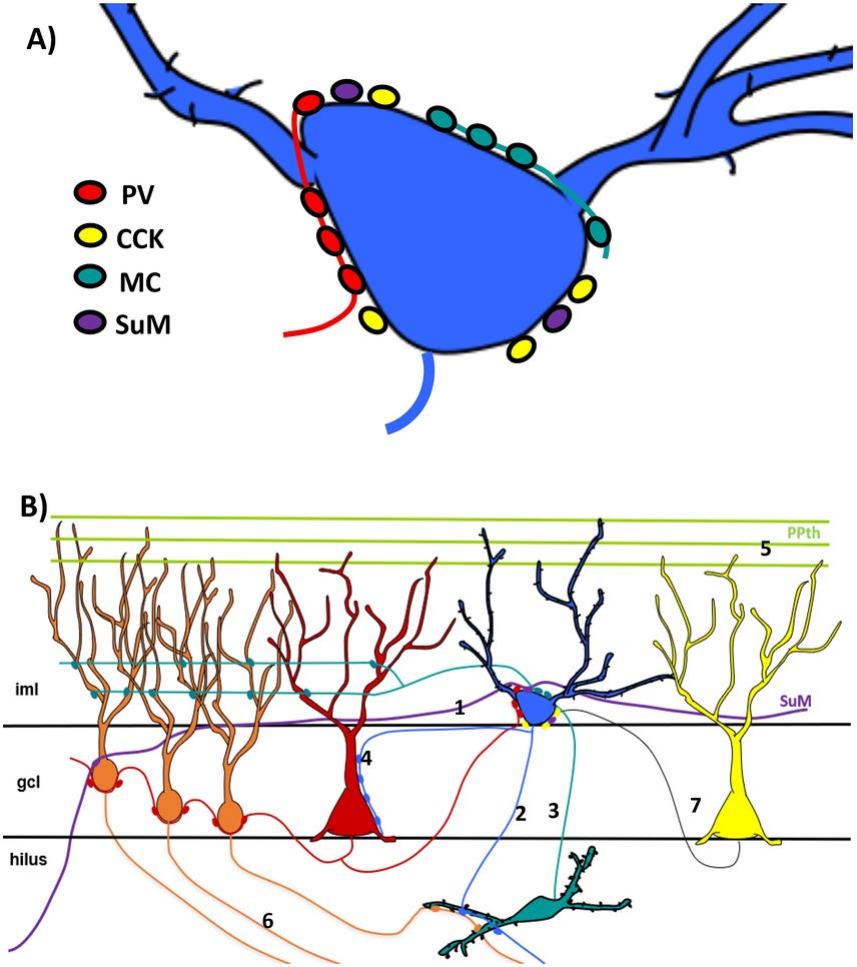
Diagrams showing the perisomatic innervation of semilunar granule cells and outer molecular granule cells. **(A)** Both, semilunar granule cells and outer molecular granule cells are perisomatically innervated by axons from the mossy cells (MC), the supramammillary nucleus (SuM), fast-spiking basket cells (PV), and regular-spiking basket cells (CCK). **(B)** Semilunar granule cells are hyperpolarized by the projections from the supramammillary nucleus (1). When semilunar granule cells fire, they project onto mossy cells (2) that in turn project back onto the soma of semilunar granule cells (3) producing a state of recurrent excitation (hilar upstate). Semilunar granule cells also project onto fast-spiking basket cells (4) that project back onto semilunar granule cells, likely timing their firing. Mossy cells and fast-spiking basket cells also project onto normal granule cells, allowing the latter to become more excitable. This allows excitation from the entorhinal cortex (5) to pass through to CA3 (6). Regular spiking basket cells also contribute to modulating the firing of semilunar and basket cells (7). Outer molecular layer granule cells may facilitate the dentate gyrus in a similar manner; however, their engagement with mossy cells and contribution to the perisomatic innervation of fast-spiking basket cells remains unknown. The cells in the diagram represent the different types of cells involved in the circuitry and do not necessarily represent either that they are all located at the same level in the dentate gyrus or in immediate proximity. Iml, inner molecular layer; oml, outer molecular layer.

The abundance of perisomatic boutons on these cells (both inhibitory and excitatory) as well as the occasional presence of somatic spines on them and the fact that they often express Fos are indicative of their level of activity. Normal granule cells are also innervated by these afferents, but mossy cells target them on the dendrites rather than on the soma, whereas supramammillary fibers do not extend to all the granule cell layer. Overall, normal granule cells have low excitability (Lopez-rojas and Kreutz, 2016). However, the fact that semilunar granule cells are intrinsically more excitable, and are involved with mossy cells in the hilar up-states (Afrasiabi et al., 2022; Larimer and Strowbridge, 2010), and innervate selectively the soma of local parvalbumin basket cells (Rovira-Esteban et al., 2020), combined with the data of the present study, suggests that they play a critical role in both the activation and the synchronization of the granule cells in the granule cell layer necessary for the processing the information of the entorhinal cortex (Figure 12). The fact that they are also strongly innervated by the supramammillary nucleus could indicate that this nucleus can greatly influence the availability of the semilunar granule cells to excitation. Semilunar granule cells receive more frequent spontaneous excitatory synaptic inputs than granule cells (Dovek et al., 2025). The excitatory perisomatic contacts from mossy cells and supramammillary nucleus described in this study may play a role in this phenomenon. The present results provide a basis for considering semilunar granule cells as the neuroanatomical substrate through which this control is exerted.

### Perisomatic innervation of outer molecular layer granule cells

The results for outer molecular granule cells were qualitatively similar to those for semilunar granule cells. These cells are innervated by inhibitory fast-spiking and regular-spiking basket cell contacts, as well as excitatory supramammillary nucleus and mossy cell contacts.

Inhibitory innervation was weaker for parvalbumin boutons when compared to semilunar granule cells, but inhibition by CB1r was similar to semilunar granule cells.

They also received boutons from mossy cells. However, since we could not quantify the number of calretinin boutons on semilunar granule cells, we cannot compare these two populations of granule cells. This is surprising, given that these cells are not in areas where mossy cell fibers have been described, that is, the inner molecular layer. Innervation from mossy cells is necessary for the function and maturation of granule cells (Marqués-Marí et al., 2007). The presence of mossy cell fibers in the outer molecular layer that specifically target granule cells in this area demonstrates the specificity of this connection and its relevance to the function of outer molecular layer granule cells.

Similarly, it was unexpected that these granule cells would be contacted by supramammillary boutons. Supramammillary boutons have mostly been described in the upper side of the granule cell layer and in CA2. The existence of specific supramammillary fibers that innervate granule cells in this area demonstrates the importance of this connectivity for the function of these cells. When comparing the number of supramammillary boutons to semilunar granule cells, the innervation was weaker but relevant.

The outer molecular layer granule cells are perisomatically innervated by the same afferents as the semilunar granule cells, though they receive contacts from fewer parvalbumin and supramammillary boutons. They also have somatic spines. Therefore, they strongly resemble semilunar granule cells. However, it is unclear whether they engage in hilar up-states with mossy cells or target parvalbumin cells in the granule cell layer. The number of granule cells in the outer molecular layer is very small compared to semilunar granule cells, and the innervation is weaker. We can only speculate that these cells may play a role similar to that of semilunar granule cells.

### Study limitations

We could not properly evaluate the number of boutons from mossy cells onto semilunar granule cells. The density of calretinin boutons in the inner molecular layer is very high (Blasco-Ibáñez and Freund, 1997) and literally engulfs the cells that have their soma in this layer. In addition, these calretinin boutons are small; they also contact dendrites of the granule cells in the inner molecular layer (Blasco-Ibáñez and Freund, 1997). Confocal scanning microscopy does not allow us to ascertain whether a bouton is making synaptic contact or is just in apposition. Electron microscopy allowed us to confirm that there were abundant calretinin boutons making synaptic contacts on the semilunar granule cells, but calculating the number of boutons will require the complete reconstruction of the whole cells in ultrathin sections. Also, the poor penetration of the calretinin antibody in this area with aldehyde-fixed materials would hamper the process.

Another limitation is that we could not obtain similar data for normal granule cells, which would allow us to compare with molecular layer granule cells. Granule cells are densely packed in the granule cell layer, often with no neuropil between them. This extreme compaction makes the study of the perisomatic contacts under confocal scanning on the whole surface of the soma impossible. The combination of the postsynaptic markers gephyrin and PSD 95 (data not shown) did not help enough. First, the penetration for these markers was insufficient to evaluate the whole cell, and then, for granule cell layer granule cells, the technique did not offer spatial resolution enough. These factors can explain the comparative lack of information on the quantification of the perisomatic innervation of the granule cells in the literature. Evaluation of electron microscopy allowed us to confirm that fast spiking basket cells and regular spiking basket cells contacted the granule cells in the granule cell layer as previously described (Freund and Buzsáki, 1996). Supramammillary boutons contacted perisomatically normal granule cells in the upper part of the granule cell layer, but were scarce in the central part, as described in rats (Maglóczky et al., 1994). Mossy cell boutons do not seem to contact perisomatically normal granule cells when they pass through the granule cell layer despite contacting the proximal dendrites of normal granule cells in the inner molecular layer (Blasco-Ibáñez and Freund, 1997). Using transmission electron microscopy to evaluate the number of boutons on normal granule cells would have been difficult because of incomplete penetration of the markers and the number of sections to consider. Another question to consider is that likely normal granule cells are not a homogeneous population, as many different immunostainings show; for example, supramammillary nucleus afferents contact normal granule cells in the upper border of the granule cell layer but are scarce at other levels.

## Conclusion

The perisomatic innervation of semilunar granule cells is well-developed. Both inhibitory and excitatory perisomatic contacts are present. Due to their unique characteristics, semilunar granule cells appear to play a pivotal role in controlling the synchronization and excitation of granule cells and the input of entorhinal cortex information into the hippocampus. The relevance of supramammillary input to semilunar granule cells provides a morphological basis for the supramammillary nucleus’s facilitating effect on the dentate gyrus. Granule cells in the outer molecular layer receive the same afferents on the soma, although there are fewer perisomatic boutons. The lack of information on these cells makes it difficult to determine whether they should be considered alongside the semilunar granule cells.

## Data Availability

The raw data supporting the conclusions of this article will be made available by the authors, without undue reservation.

## Supplementary material

Supplementary Figure 1 Thy1 neurons in the inner and outer molecular layer are granule cells. Thy1 is stochastically expressed in principal cells. In the dentate gyrus, they are found in all layers. Colocalization with Prox1 shows that those Thy1 cells in the inner molecular layer **(A)**, the lower part of the outer molecular layer **(B)** or higher, in the outer molecular layer **(C)** are granule cells. Gcl, granule cell layer; iml, inner molecular layer; oml, outer molecular layer. Scale bar 50 μm.

Supplementary Figure 2 Calretinin does not label Vglut2 boutons in mouse. In rats, supramammillary boutons labeled with Vglut2 colocalize with calretinin **(A)**, but in mice, they do not **(B)**. Therefore, calretinin can be used as a specific marker for mossy cell axons in mice. This rules out the possibility that the calretinin perisomatic contacts originate in the supramammillary nucleus. CR, calretinin; gcl, granule cell layer; h. hilus, iml, inner molecular layer. Scale bar 50 μm.

Supplementary Figure 3 Semilunar granule cells and outer molecular layer granular cells are contacted by supramammillary nucleus boutons using anterograde transport. 10 KDa BDA in the supramammillary nucleus **(A)**, shows anterogradely labeled boutons in the supra-granular layer of the dentate gyrus **(B)**. When combined with Prox1, both semilunar granule cells and outer molecular layer granule cells were surrounded by perisomatic labeled boutons **(C,D)**. The analysis of the cell in **(D**, arrow, asterisk**)** under electron microscopy reveals that these boutons make asymmetric contacts with the cell **(E–G**, arrows**)**. DG, dentate gyrus; gcl, granule cell layer; h. hilus; iml, inner molecular layer; oml, outer molecular layer; omlGC, outer molecular layer granule cell. Scale bar 500 nm.

Supplementary Figure 4 Granule cells in the molecular layer are contacted by cholecystokinin boutons in rat. A granule cell labeled with Prox1 is targeted by cholecystokinin boutons in the outer molecular layer of the rat dentate gyrus **(A**, asterisk**)**. The correlation of the cell under electron microscopy **(B)** shows that the cell was contacted by large boutons making symmetric contacts **(C,D**, arrows**)**. CCK, cholecystokinin; gcl, granule cell layer; iml, inner molecular layer; oml, outer molecular layer. Scale bar 500 nm.
