# Supplementary figures and images for: Perisomatic innervation on the semilunar granule cells and outer molecular layer granule cells of the dentate gyrus of the mouse
Source: Front Neuroanat. 2026 Apr 29;20:1749335. doi: 10.3389/fnana.2026.1749335 (PMC13168021; doi:10.3389/fnana.2026.1749335)

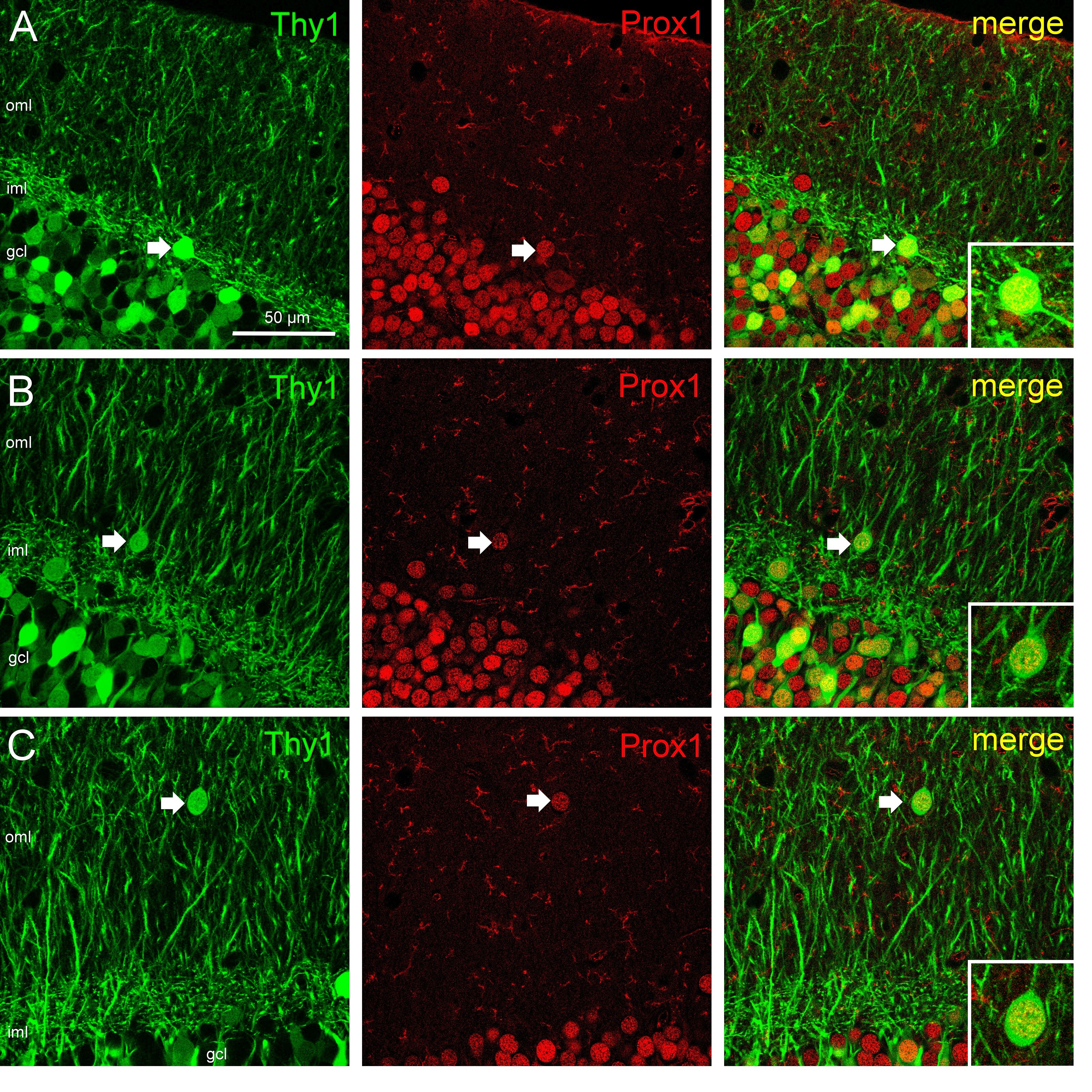

Supplement: Supplementary Figure 1 — Thy1 neurons in the inner and outer molecular layer are granule cells. Thy1 is stochastically expressed in principal cells. In the dentate gyrus, they are found in all layers. Colocalization with Prox1 shows that those Thy1 cells in the inner molecular layer (A), the lower part of the outer molecular layer (B) or higher, in the outer molecular layer (C) are granule cells. Gcl, granule cell layer; iml, inner molecular layer; oml, outer molecular layer. Scale bar 50 μm. [file Image_1.JPEG]

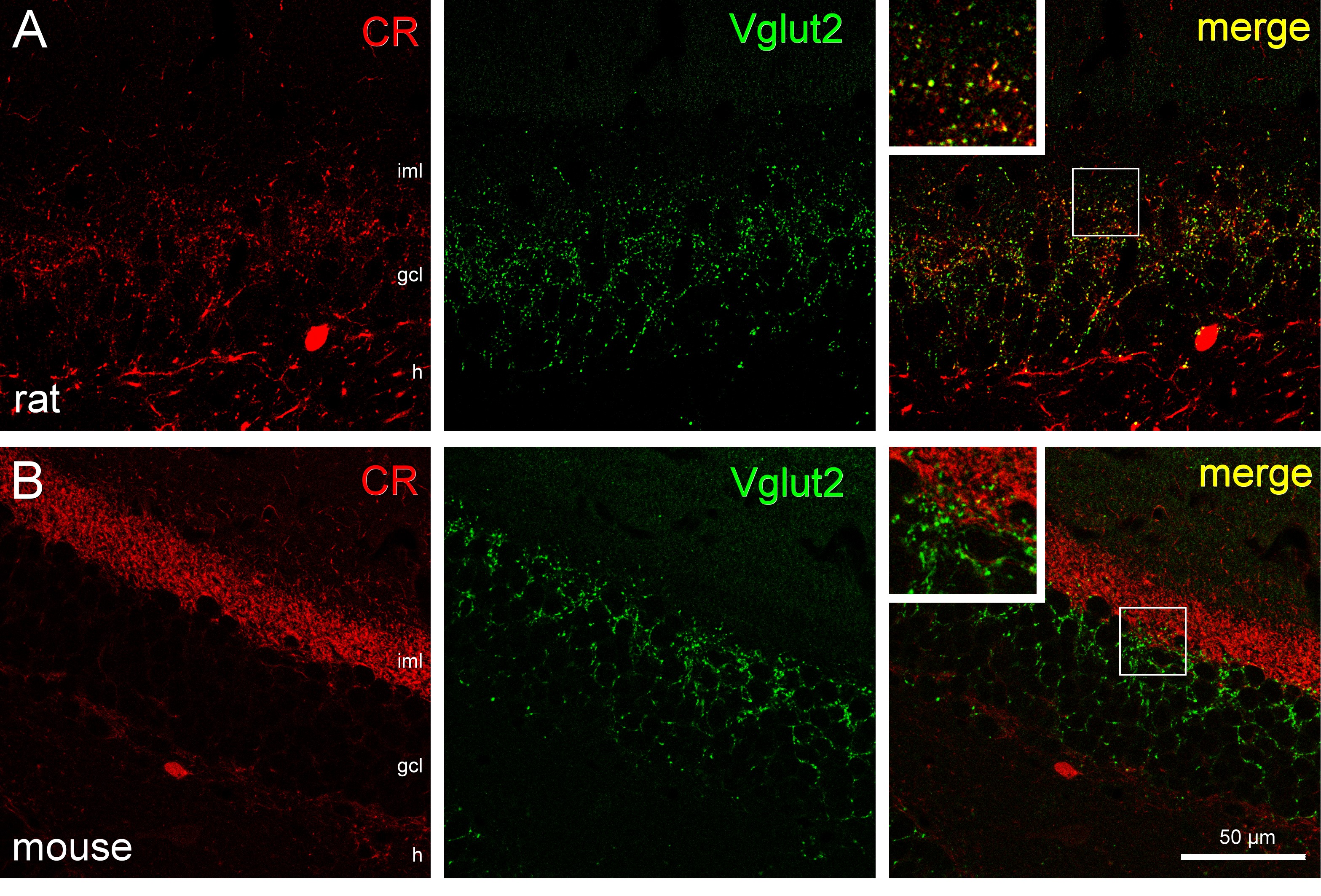

Supplement: Supplementary Figure 2 — Calretinin does not label Vglut2 boutons in mouse. In rats, supramammillary boutons labeled with Vglut2 colocalize with calretinin (A), but in mice, they do not (B). Therefore, calretinin can be used as a specific marker for mossy cell axons in mice. This rules out the possibility that the calretinin perisomatic contacts originate in the supramammillary nucleus. CR, calretinin; gcl, granule cell layer; h. hilus, iml, inner molecular layer. Scale bar 50 μm. [file Image_2.JPEG]

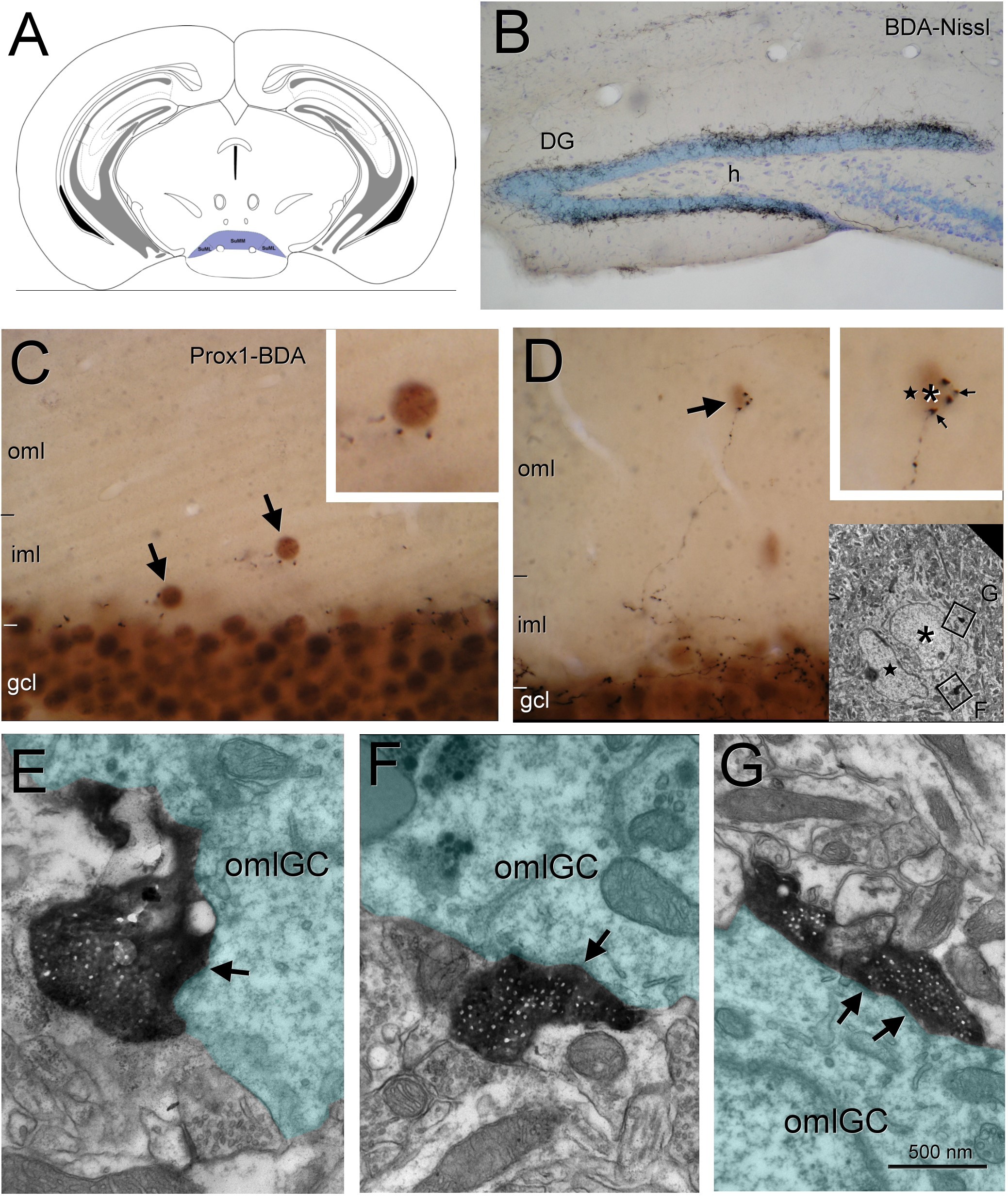

Supplement: Supplementary Figure 3 — Semilunar granule cells and outer molecular layer granular cells are contacted by supramammillary nucleus boutons using anterograde transport. 10 KDa BDA in the supramammillary nucleus (A), shows anterogradely labeled boutons in the supra-granular layer of the dentate gyrus (B). When combined with Prox1, both semilunar granule cells and outer molecular layer granule cells were surrounded by perisomatic labeled boutons (C,D). The analysis of the cell in (D, arrow, asterisk) under electron microscopy reveals that these boutons make asymmetric contacts with the cell (E–G, arrows). DG, dentate gyrus; gcl, granule cell layer; h. hilus; iml, inner molecular layer; oml, outer molecular layer; omlGC, outer molecular layer granule cell. Scale bar 500 nm. [file Image_3.JPEG]

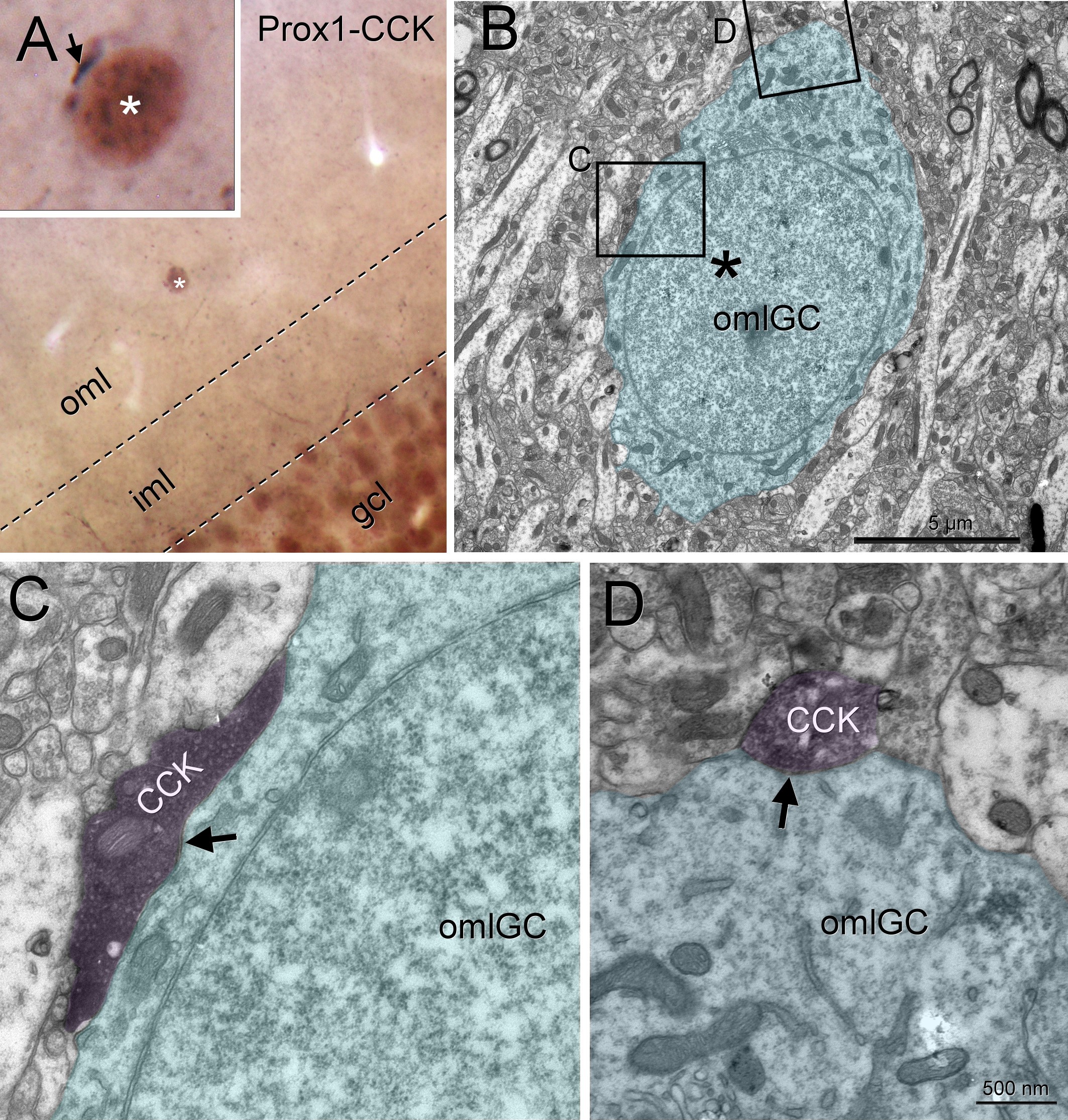

Supplement: Supplementary Figure 4 — Granule cells in the molecular layer are contacted by cholecystokinin boutons in rat. A granule cell labeled with Prox1 is targeted by cholecystokinin boutons in the outer molecular layer of the rat dentate gyrus (A, asterisk). The correlation of the cell under electron microscopy (B) shows that the cell was contacted by large boutons making symmetric contacts (C,D, arrows). CCK, cholecystokinin; gcl, granule cell layer; iml, inner molecular layer; oml, outer molecular layer. Scale bar 500 nm. [file Image_4.JPEG]
